# The Regulation of Pyroptosis and Ferroptosis by MicroRNAs in Cardiovascular Diseases

**DOI:** 10.31661/gmj.v12i.2933

**Published:** 2023-08-30

**Authors:** Akram Shariati, Venus Shahabi Raberi, Mehdi Masumi, Ali Tarbiat, Elham Rastgoo, Reza Faramarz Zadeh

**Affiliations:** ^1^ Department of Cardiology, School of Medicine, Urmia University of Medical Sciences, Urmia, Iran; ^2^ Seyed-Al-Shohada Cardiology Hospital, Urmia University of Medical Sciences, Urmia, Iran; ^3^ Department of Radiology, School of Medicine, Shiraz University of Medical Sciences, Shiraz, Iran

**Keywords:** Heart Failure, Cardiovascular Diseases, MiRNAs, Pyroptosis, Ferroptosis

## Abstract

Cardiovascular diseases (CVDs) are considered the most prevalent noncommunicable disease and the leading cause of death worldwide. A plethora of evidence has revealed that microRNAs (miRNAs) could control the inhibition or progression of CVDs by regulating pivotal cell processes ranging from metabolism and homeostasis to programmed cell death (PCD). Pyroptosis and ferroptosis are two major types of nonapoptotic PCDs involved in the pathogenesis of heart failure. However, no study has discussed the crosstalk between miRNAs and these two types of PCDs in the CVDs. The current review demonstrated that different types of miRNAs can regulate both ferroptosis and pyroptosis and thereby affect CVDs progression and inhibition. Altogether, the discussed content encourages further studies to confirm that mentioned pathways are suitable to be considered as novel therapeutic approaches against CVDs.

## Introduction

Cardiovascular diseases (CVDs) have been described as one of the most substantial health concerns and the most prevalent noncommunicable disease all around the world [1][2]. According to recent statistics, CVDs are considered the leading cause of morbidity and mortality, accounting for over 30% of all deaths worldwide [3]. More substantially, it is well-documented that the mortality from CVDs continues to increase; according to 2012 statistics. The reported death rate was 17.5 million which rose to about 18 million in 2016. Moreover, CVDs mortality is expected to reach more than 22 million deaths by 2030 [4]. A plethora of evidence documented that CVDs refer to all types of disorders associated with the cardiac tissue and blood vessels, including cardiac arrhythmias, atrial fibrillation, myocardial fibrosis, cerebrovascular disease, peripheral vascular disease, deep vein thrombosis, hypertension, atherosclerosis, pulmonary embolism, and heart diseases such as coronary heart disease, hypertrophic cardiomyopathy, pericarditis, dilated cardiomyopathy, congenital heart disease, diabetic cardiomyopathy, and rheumatic heart disease all of which can result in a drastic condition known as heart failure [4][5][6]. Heart failure is recognized as a heterogeneous syndrome with complicated case detection [7]. The incidence and prevalence of heart failure is increasing worldwide due to an aging population. Moreover, heart failure is considered the most common causative factor for hospitalization in people over the age of 65 [8].

Furthermore, it has been anticipated that approximately 8 million people over the age of 18 could suffer from heart failure, and it was estimated that approximately 40 million patients are currently affected [9]. The majority of the human genome, about 98%, consists of noncoding DNA, formerly known as junk DNA. Nevertheless, the current findings suggest that almost three-quarters of the genome potentially could be wiped out. This transcriptional potential demonstrates both the significance and benefits of noncoding RNAs in the understanding, diagnosing, and treating a variety of human complications [10].

In fact, noncoding RNAs are the major modulators regulating a variety of crucial cellular processes including cellular growth, development, differentiation, and death, hence considered to determine cell fate. Consequently, any impairment in their physiological transcription is associated with serious disorders [11][12]. Recently, a large body of evidence has addressed the role of noncoding RNAs in the etiology of CVDs [13]. Therefore, the current study aimed to discuss the regulation of programmed cell death (PCD) processes including ferroptosis and pyroptosis by microRNAs and their role in the pathophysiology of heart failure.

MicroRNAs (miRNAs) As the Major Regulators of Vital Cell Processes

MiRNAs are a well-described class of short noncoding RNAs (snRNAs) with 19-24 nucleotides in length (average of 22 nucleotides) recognized as the main regulators of cellular gene expression [14]. In the early 90s, two distinct studies from Ambros and Ruvkun groups reported the first miRNA, called lin-4, in Caenorhabditis elegans [15][16]. Nonetheless, fundamental information regarding biosynthesis, function, and mechanism was elucidated during the following decades. Moreover, the ongoing identification of subsequent miRNAs, is still being elucidated and novel miRNAs are being identified and described. The majority of miRNAs are directly transcribed by RNA polymerase II from DNA sequences into primary miRNAs. After processing, the primary miRNAs are turned into precursor miRNAs and mature miRNAs [17]. Conventionally, scientists have recognized miRNAs as negative regulators of gene expression, which are often mediated by the direct interaction of miRNAs with the 3′ UTR of target mRNAs, 5′ UTR, and coding sequences to suppress expression via the degradation of mRNA or silencing of the gene. However, recent findings demonstrated that these types of snRNAs can induce overexpression of genes under specific conditions [18]. The mentioned less-indicated feature of miRNAs appears to be mediated by particular interactions with gene promoters [19].

The Mechanisms of MiRNA-mediated Regulation of Gene Expression

A variety of mechanisms have been described for miRNA-regulated gene expression, including the intranuclear miRNA-mediated transcriptional and posttranscriptional gene expression [20], miRNA-mediated gene silencing via the minimal miRNA-induced silencing complex (miRISC) [21], and miRNA-mediated translational activation [22]. Regardless of the nature of the mechanism by which miRNAs modify gene expression, these snRNAs are considered crucial for major cellular processes including cellular development, proliferation, differentiation, and death. In addition, miRNAs are involved in a plethora of pivotal biological processes. Therefore, impaired miRNAs expression has been attributed to various diseases.

Moreover, the secretion of miRNAs into extracellular fluids, which have been extensively documented as promising biomarkers of a variety of disorders, is another important perspective of the beneficial function of miRNAs. Furthermore, miRNAs appeared to contribute to cell-cell communications as signaling molecules [23][24].

MiRNAs Represent a Prominent Role in CVDs

The diagnostic benefits of miRNAs as biomarkers in CVDs for specific disease entities, including myocardial infarction, coronary artery disease, and heart failure have been investigated in various experimental studies and patient cohorts. The outcomes of these attempts have put miRNAs on the verge of implementation in clinical disease assessment [25][26]. Since microRNAs can affect a variety of cellular pathways, including metabolic and energy homeostasis, which are highly involved in CVDs pathogenesis, it is logical to assume a clinical relevance between miRNAs and CVDs [27]. It is documented that miRNAs are extensively involved in vascular integrity and endothelial cell function as miRNAs contribute to different stages of plaque progression as well as any dysregulation in miRNAs is associated with the destabilization and rupture of atherosclerotic plaques [28].

Moreover, several miRNAs known as angiomiRs, including miR-210, miR-222, miR-126-3p, miR-221, mir-92a, and miR-132 are responsible for the regulation of angiogenic processes [29]. miR-126-3p is considered a major regulator maintaining vascular integrity and functions as a pro-angiogenic factor [30].Furthermore, miR-126-3p is present in platelets and functions as the modulator of platelet aggregation since its inhibition attenuates platelet aggregation [31]. In addition, it is reported that the levels of miR-1, miR-126-3p, and miR-208 in coronary artery disease and myocardial infarction were increased, while the levels of miR-21, miR-133, and miR-195 were decreased [32][33].

Similarly, several reports have attributed heart failure to the dysregulation of miRNAs. Heart failure at the cellular level results from the dysfunction of cardiomyocytes and their fibrosis due to excessive extracellular matrix accumulation [34]. miR-133 is highly expressed in cardiomyocytes; however, in patients with hypertrophic cardiomyopathy, the level of expression reduces significantly [35].

In addition, miR-1, a part of the same cluster as miR-133, is expressed abundantly in cardiomyocytes, whereas lower levels are reported in patients with heart failure [36].

Interestingly, either the decrease or the increase in miR-1 levels has been associated with electrophysiological abnormalities [37]. miR-208, being responsible for the regulation of the balance between the α- and β-myosin heavy chains, is highly abundant in cardiomyocytes. It is documented that the modification of miR-208 is followed by better cardiac function in patients with heart failure [34][38]. Furthermore, the increased and repressed expression of miR-25 is reported in failing human hearts [39].

The Crosstalk between MiRNAs and PCDs in the Incidence of Heart Failure

The role of PCDs as well as miRNAs dysregulation in CVDs, and on the other hand the regulation of PCDs by miRNAs characterize the crosstalk between miRNAs and PCDs in the incidence of CVDs. Despite the wide variety of cell death processes, previous studies have divided these processes into two major categories, including accidental cell death programs and PCDs.

Accidental cell death programs have been described as a passive process in that uninspected necrosis is the main type, whereas PCDs are active processes consisting of two major subtypes, including apoptotic and non-apoptotic programs [40][41]. Necroptosis, pyroptosis, autophagy, and ferroptosis are nonapoptotic PCDs each with distinct biochemical, morphological, and functional features [42]. Since PCDs function in important cellular processes including the maintenance of homeostasis and drastic events including the incidence of disorders, the researchers are keen to clarify the association of PCDs with human health problems. MiR-150, for example, can inhibit the death of cardiomyocytes during cardiac injury and thereby protect against heart failure [43]. The inhibition of autophagy by miR-221 is followed by the promotion of heart failure via modulating the p27/CDK2/mTOR axis [44].

Contradictory, the inhibition of autophagy by miR-30 resulted in the cardioprotection against heart failure caused by doxorubicin [45], an extensively applied chemotherapeutic agent. In addition, miR-30 and miR-132 can regulate cardiomyocyte apoptosis in heart failure [46][47].

Although there have been extensive studies on the crosstalk between miRNAs and PCDs in heart failure, the regulation of pyroptosis and ferroptosis by miRNAs has not yet been fully elucidated. After briefly introducing these two non-apoptotic subtypes, the present study discusses the regulation of pyroptosis and ferroptosis by miRNAs in heart failure.

A Concise Overview of Pyroptosis

Pyroptosis is recognized as an inflammasome-induced PCD. Pyroptosis has been reported to be mediated by specific proteins known as gasdermins. The initial reports of pyroptosis occurred in 1992 in myeloid cells infected by pathogens [48]. A variety of documents have demonstrated that pyroptosis is involved pivotally in the clearance of bacterial and viral infections through two distinct but proportionate processes [49]. Pyroptosis is considered a critical process in physiological cellular functions since the dysregulation of pyroptosis is accompanied by dysfunction in the adaptive immune defenses stimulation, failed efficiency of pathogens clearance, and tissue damage [50]. In addition to infectious states, it is documented that pyroptosis is activated during other disorders, including cancer and chronic diseases [51][52].

Previous documents have characterized pyroptosis by the pore formation in the plasma membrane followed by the swelling of the cytoplasm, the rupture of the plasma membrane, and finally, the entrance of the cell content into the extracellular environment [53]. The released materials include inflammatory mediators (e.g., IL-1β); thereby, pyroptosis is believed to be associated with inflammations, whether local or systemic [51]. Pyroptosis is considered to be induced by two distinct mechanisms, including the canonical and non-canonical pathways. The canonical pathway is mediated by the caspase-1 inflammasome mechanism, and the caspase-4/5 (humans) or caspase-11 (mice) inflammasome mechanism is involved in the non-canonical pathways [54].

A Concise Overview of Ferroptosis

Ferroptosis has been described as an iron-dependent type of PCD. Two major biochemical markers including the accumulation of iron and lipid peroxidation, are considered the characteristics of ferroptosis. The accumulation of iron, due to its redox ability, leads to the generation of excessive free radicals, damaging DNA, and disrupting the DNA repair system, all of which are followed by the acceleration of cell senescence known as ferritin aging [55]. Hence, the excessive accumulation of Fe2+ has considered being an early signal of ferroptosis, and its overload is involved in the pathogenesis of a variety of human diseases [56]. The removal of lipid electrons in the plasma membrane by free radicals is known as lipid peroxidation. Lipid peroxidation is followed by the overproduction of reactive oxygen species, oxidation of membrane polyunsaturated fatty acids (PUFAs), and formation of LOOH. It is documented that PUFAs are involved in pathological processes such as DNA damage, pro-inflammatory, and the activity of cellular enzymes as well as is known as a cell death signal in different types of PCDs, including apoptosis, autophagy, and ferroptosis [57][58]. Indeed, ferroptosis is mediated directly by damaged PUFAs [59].

The Crosstalk between MiRNAs and Pyroptosis and Ferroptosis in Heart Failure

Similar to those noted for the contribution of miRNAs in the incidence of heart failure, a variety of studies have documented the involvement of pyroptosis [60][61] and ferroptosis types of PCD processes in the pathogenesis of CVDs.

In atherosclerosis, characterized by abnormal deposition of lipids in the aorta and obstruction of blood flow leading to coronary heart disease and stroke, inflammatory responses and different types of immune cells are involved [62][63]. Hence, pyroptosis has been shown to contribute to the formation and progression of atherosclerosis via the promotion of the release of inflammatory mediators and the stability of the plaque [64]. In addition, the association between pyroptosis-related inflammasomes and warning factors for atherosclerosis related to lipid metabolisms such as cholesterol crystals and oxidized low-density lipoprotein clarify the involvement of pyroptosis in the progression of heart failure [65]. Along with that, elevated levels of inflammatory and lipid mediators result in the induction of pyroptosis in vascular endothelial cells [66][67].

The overproduction of free radicals induced by hyperglycemia is followed by the activation of pyroptosis-related inflammasomes, alteration in lipid metabolism and energy expenditure, and finally, pyroptosis in cardiomyocytes [68][69]. Furthermore, pyroptosis has been demonstrated to be involved in the cardiac fibroblasts and cardiac smooth muscle cells as well as in the pathogenesis of cardiac hypertrophy [70]. Similarly, various studies have documented the association of ferroptosis with CVDs. In hypertrophic cardiomyopathy, for example, iron overload, along with excessive production of free radicals and elevated levels of lipid peroxidation leads to ferroptosis death of cardiomyocytes [71][72]. In addition, alteration in the levels of factors associated with energy homeostasis including lactate and glucose is followed by the induction of ferroptosis in hypertrophic cardiomyopathy [73]. Induction of ferroptosis in dilated cardiomyopathy has been evidenced under similar conditions as in hypertrophic cardiomyopathy [74]. In addition, previous studies have revealed the involvement of ferroptosis in heart failure, suggesting this cell death process is a promising therapeutic strategy [75][76].

Despite extensive evidence for the involvement of both miRNAs and cell death processes in pyroptotic and ferroptotic pathways in heart failure, no study has addressed the possible crosstalk. Interestingly, studies assessing the regulation of pyroptosis by miRNAs in cardiomyocytes have been generally related to cardiac complications caused by diabetes and hyperglycemia. In the cardiomyocytes of diabetic models, miR-9 could inhibit pyroptotic-dependent cardiac cell loss via attenuation of hyperglycemia-induced ELAVL1 upregulation; hence miR-9 could suppress heart failure in diabetics through the inhibition of pyroptosis [77].

In addition, it is documented that miR-214-3p reduced the activity of caspase 1 and thereby alleviated pyroptosis in high glucose-induced cardiomyopathy [78]. Furthermore, miR‑141‑3p suppressed high glucose‑induced pyroptosis and prevented diabetic cardiomyopathy [78]. Concordantly, miR-133a-3p targeted IKKε, suppressed pyroptosis, and attenuated cardiomyocyte hypertrophy [79]. However, in high glucose-induced cardiomyopathy, miR-30d targeted foxo3a, reduced apoptosis recruitment domain, and increased caspase 1 and inflammatory markers leading to pyroptosis of cardiomyocytes and heart failure [80]. On the contrary, the studies that have determined the crosstalk between ferroptosis and miRNAs have been more diverse. An interesting microarray data analysis demonstrated the association of various miRNAs with genes involved in ferroptosis in dilated cardiomyopathy and hypertrophic cardiomyopathy [81]. Similarly, a bioinformatics analysis reported the interaction of different miRNAs, particularly miR-21-3p and miR-1892, with ferroptosis-related genes in septic cardiomyopathy [82].

Another bioinformatic analysis revealed that in calcific aortic valve disease, a prevalent state culminating in aortic stenosis and heart failure, several miRNAs are related to key ferroptosis genes including CYBB, HMOX1, and HIF-1α [83].

On the other hand, the modification in the levels of glutathione peroxidase 4 by miR-375-3p resulted in the acceleration of ferroptosis, leading to the promotion of cardiac fibrosis [84]. In addition, the sponging of miR-150-5p is associated with attenuation of ferroptosis and activation of CCND2 all of which lead to the alleviation of diabetic cardiomyopathy [85]. Importantly, all the mentioned studies, both those examining the regulation of pyroptosis by miRNAs and those that assessed the regulation of ferroptosis by miRNAs, represented promising findings for the possible consideration of these processes as a novel therapeutic approach for CVDs. Nevertheless, the conduct of further studies to confirm the capability of these processes as a management strategy for heart failure is strongly recommended.

## Conclusion

The findings of the current review study reveal that the crosstalk between miRNAs with both ferroptosis and pyroptosis plays a significant role whether in the inhibition or the progression of CVDs. Therefore, dependent on the confirmation of further studies, they could be assumed as a promising novel management strategy for heart failure.

## Conflict of Interest

There are no conflicts of interest to declare.
